# Effectiveness of a Training Program in School Teachers on the Role of Sugars in Oral and General Health

**DOI:** 10.7759/cureus.28865

**Published:** 2022-09-06

**Authors:** Upendra S Bhadauria, Bharathi M Purohit, Deepali Agarwal, Ritu Duggal, Vijay Mathur, Harsh Priya

**Affiliations:** 1 Dentistry, All India Institute of Medical Sciences, New Delhi, IND

**Keywords:** school teachers, oral health, oral health promotion, health promotion, health education

## Abstract

Background: Schools have a powerful influence on children's development and well-being, and school teachers are considered role models to transmit life values and instill health-related behaviors. The effectiveness of a training program for school teachers in understanding the role of sugars and their impact on oral and overall health has not been previously reported.

Aim: The present study aimed to assess the effectiveness of a training program for school teachers on the role of sugars in oral and general health.

Methods: An interventional study was carried out on 308 Kendriya Vidyalaya Sangathan school teachers to evaluate the effectiveness of a training program in improving school teachers' knowledge of the role of sugars in oral and overall health. The training program was carried out using a training module, and a validated questionnaire was utilized to assess school teachers' knowledge before and after the training program.

Results: The overall evaluation of mean knowledge change scores revealed significantly higher scores after the training program (8.12 ±1.58) when compared with the pre-training scores (6.84±1.66)

Conclusion: A training program on the role of sugars in oral and general health effectively improved school teachers' knowledge.

## Introduction

The sugars form an essential component of our diet and serve as a quick and easy fuel source for the body. Poor diet, high sugar consumption, and physical inactivity are some of the most common causes of non-communicable diseases (NCDs). NCDs are considered one of the leading causes of death, a significant proportion of which is seen in low and middle-income countries. A high level of free sugar intake is thus of concern because of its association with poor dietary quality, obesity, and risk of NCDs. The association between intake of free sugars and dental caries, particularly with dental diseases being the most prevalent NCDs globally [1].

The WHO Guideline on sugars intake recommends reducing the intake of free sugars in children and adults, which should not exceed 10% of the total energy intake. To protect oral health throughout life, WHO also suggested a further reduction to below 5% of total energy intake (as a "Conditional Recommendation"). The guidelines further recommended that the intake should not be increased for populations currently consuming low levels of sugars [2]. Understanding the impact of sugars on oral and overall health and the prevention measures at a young age thus becomes of utmost importance, especially with the knowledge that dietary risk factors can be modified. The burden of Non-communicable diseases can also be reduced if positive health-related habits are incorporated early in children.

Schools profoundly influence children's development and well-being [3]. School teachers are considered role models to transmit life values and play an important role in instilling health-promoting habits at a young age that sustain lifelong and aid in establishing behaviors, beliefs, and attitudes related to health.

The different forms and chains of schools running globally cater to the educational needs of children. In addition to imparting knowledge and values, these federal systems of schools provide a unique and one-step opportunity to disseminate health and oral health-related information through school-based programs. One such organization in India that comprises approximately 14 lakh students in India is the Kendriya Vidyalaya Sangathan (KVS). The KVS is an autonomous body under the Ministry of Education, Government of India, spread across 25 regions of the country with 48314 employees and 5 Zonal Institute of Education and training [4].

School-based programs increase children's access to dental services, especially those from disadvantaged socioeconomic backgrounds. The school-based oral health programs include oral health education (OHE), tooth-brushing activities, preventive treatment programs, etc. A systematic review assessing the effectiveness of school-based oral health programs from preschool to high school has also reported positive outcomes and better oral health knowledge, behaviors, attitude, status, and quality of life [5].

School oral health programs utilizing training modules in improving school teachers' knowledge of oral health and their role in improving the oral health of school children have also been reported in the literature [5-6]. However, no previous program has focused on training modules utilizing sugars and their impact on oral and overall health. A training module highlighting the role of sugars was developed. The present study evaluated the module's effectiveness for school teachers on the role of sugars in oral and overall health.

## Materials and methods

The ethical clearance to conduct the study was obtained from the institutional ethics committee of the All India Institute of Medical Sciences (AIIMS), New Delhi (IEC-613/03.09.2021, RP-11/2021). Informed consent was obtained from the school teachers before the start of the study. An interventional study was carried out to evaluate the effectiveness of a training program in improving school teachers' knowledge of the role of sugars in oral and overall health. The Centre carried out this interventional study for Dental Education and Research (CDER), AIIMS, New Delhi, in collaboration with the Kendriya Vidyalaya Sangathan India. The school teachers of KVS are divided into three groups, i.e., Primary School Teachers (PRTs), Trained Graduate Teachers (TGTs), and Post Graduate Teachers (PGTs), depending upon the classes they teach (PRTs - Grade 1st -5th, TGTs - Grade 6th -10th and PGTs - Grade 11th -12th), and school teachers from all the three groups were included in this study. The training program was carried out in coordination with the Ministry of Education and the Government of India, and the training division of the KVS nominated the teachers. The Centre developed the training module for Dental Education and Research, AIIMS- New Delhi.

Development of training module

The training module consisted of a virtual online presence through a webinar of approximately 45 minutes - 1 hour on the role of sugars in oral and general health. The content search for the development of the module was carried out by evaluating the existing curriculum and identifying the scope of integration. The training module was developed specifically for different groups of school teachers, considering the comprehension of the students they teach and in line with the existing curriculum. The presentation highlighted the role of sugars in daily life, their good and bad effects, the etiology of oral and systemic diseases from high sugar intake, and their prevention. The vetting of the developed modules was carried out by experts belonging to diversified fields with masters in health, oral health, diet, and department of communication. A penultimate version of the modules was developed incorporating feedback from the experts, which was resent to the experts for final approval. Ultimately, the final version of the training module was prepared.

Training sessions

The online training sessions were channelized as a single-day program at the Centre for Dental Education and Research, AIIMS New Delhi. The sessions were carried out in different groups from August to October 2021. The vetted modules were utilized for the training program, which was subsequently followed by a comprehensive discussion on the role of sugars. All the queries of the participants were addressed during the discussion. The teachers were administered a pre-and post-assessment questionnaire, and the effectiveness of the training module was evaluated with a post-evaluation questionnaire sent after the completion of the training module. The participants completing the pre and post-questionnaire were considered in the final analysis.

Questionnaire development

The questionnaire development was carried out, considering the training module for the specific group of teachers. The questionnaire was designed in English and developed in Google document format to assess school teachers' knowledge of the role of sugars in oral and general health. The first part of the questionnaire assessed the demographic details, whereas the second part comprised multiple-choice questions on the training content.

Validation of the questionnaire

Six experts developed a 15-item pool, initially evaluated for the content and face validity. The questionnaire was presented to experts who were acquainted with the topic and collected after one week. Based on the professional judgment of the experts, necessary changes were made. The quantitative validation of the questionnaire was carried out using the content validity ratio. The final questionnaire thus comprised 10 questions assessing the role of sugars in oral and general health.

Reliability analysis

Post validation of the questionnaire, the Test-retest reliability analysis was carried out with 15 individuals using Pearson's correlation coefficient and internal consistency measured using Cronbach's alpha. A strong positive correlation (.88) and high internal consistency (0.94) were reported. Every question of the ten-item questionnaire was assigned a single point, thus signifying a maximum score of 10 for any participant. The participants' mean scores were used to evaluate the pre and post-training knowledge scores. The effectiveness was further evaluated by categorizing the knowledge scores of participants as poor (0-3), fair (4-6), and poor (7 and above) and assessing the pre and post-knowledge change. Initially, from the three groups, 420 school teachers were nominated to participate in the training program; however, 308 school teachers (Response rate- 73.3%) participated in the program.

Statistical analysis

The data collected was entered in Microsoft Excel and subjected to statistical analysis using Statistical Package for Social Sciences (SPSS, IBM version 20.0). The significance level was fixed at 5%, and p ≤ 0.05 was considered statistically significant. Kolmogorov- Smirnov test and Shapiro-Wilks test were employed to test the normality of data. Wilcoxon signed-rank test and Chi-square test were performed for quantitative variables.

## Results

A total of 308 school teachers (113 PRTs, 101TGTs, and 94 PGTs) participated in the training program. The mean age and years of experience among the study participants were 40.76+ 8.30 and 12.18 +8.14 years, respectively, which were higher in the PGT group compared with the other groups. Gender-wise assessment of the study participants in our study revealed female predominance (74.4%) in all three groups of school teachers (Table 1).

**Table 1:** Demographic Characteristics of study participants

Variable	Primary School Teachers		Trained Graduate Teachers			Post Graduate Teachers	
	Male N (%)	Female N (%)	Male N (%)	Female N (%)		Male N (%)	Female N (%)
Gender	26 (23)	87 (77)	21 (20.8)		80 (79.2)	32 (34)	62 (66)
Total	Males: 79(25.6) Females: 229(74.4)						
	Primary School Teachers Mean ±S.D.		Trained Graduate Teachers Mean ±S.D.			Post Graduate Teachers Mean ±S.D.	
Age	37.23±7.60		41.48±8.56			44.23±7.15	
Total	Mean Age: 40.76 ± 8.30						
Years of experience	10.86±6.35		11.54±9.31			14.45±8.32	
Total	Mean Years of Experience: 12.18 ±8.14						

Assessment of the mean knowledge change scores among the three groups of teachers revealed significantly higher post-knowledge scores in all three groups of school teachers (Table 2). The overall evaluation of mean knowledge change scores also revealed significantly higher scores after the training program (Table 3) (p-value 0.001).

**Table 2:** Evaluation and comparison of mean pre and post-knowledge scores among different groups of school teachers (s)= statistically significant

School teachers	Pre-Training Knowledge Score Mean ± S.D.	Post Training Knowledge Score Mean ± S.D.	p-value
Primary School Teachers	7.39 ±1.45	8.62 ±1.24	0.001 (s)
Trained Graduate Teachers	6.56 ±1.51	7.76 ±1.45	0.001 (s)
Post Graduate Teachers	6.46 ±1.87	7.90 ±1.91	0.001 (s)

**Table 3:** Evaluation and comparison of overall mean pre and post-knowledge scores among different groups of school teachers (s)= statistically significant

Parameter	Pre-Training Knowledge Score Mean ±S.D.	Post Training Knowledge Score Mean ±S.D.	p-value
Knowledge Scores	6.84 ±1.66	8.12 ±1.58	0.001(s)

## Discussion

The present study explores the role of school teachers in reducing sugar intake among school children through a training module. Although acquiring proper knowledge to improve or maintain one's health is a complex and lengthy causal pathway, the current study further strengthened the role of training modules in school-based oral health promotional programs. This study was carried out to understand that school teachers can instill positive health-related behaviors in children with their knowledge and qualification.

One point of consideration in designing any health education program is the age group of the target population. The intervention module in the present study was specifically designed to cater to the school teachers' understanding and the targeted school children. Moreover, studies in the past have reported that oral health education given not only to the children but also to the teachers and parents will encourage children to adopt good oral health behaviors [5].

The curriculum-based training program was carried out for three groups of school teachers. The gender-wise assessment revealed that most participants were females, similar to previous studies conducted [7-8]. The more significant number of female participants in most studies may be attributed to the greater female gender predilection in the teaching profession. 

The study's findings were consistent individually in all three teacher groups, revealing an increase in the knowledge score. An overall evaluation and comparison of the knowledge change scores in the present study also revealed an increase in the post-knowledge scores following the training module. The findings of the study are consistent with the study conducted by Nyandindi U et al., [9], Jain S et al., [6], Ramroop et al., [10], Conrado CA et al., [11], Maheswari UN et al., [12], Fernando S et al., [13], Chandrashekar BR et al., [14] who also reported an increase post-training program; however, in contrast, Suwargiani AA. et al. [15] reported no impact of initial oral health training on teachers' knowledge, attitudes, and actions change in their study.

The improvement in knowledge scores attributed to the training program may not necessarily lead to behavior changes but are indicative of the role of school teachers as a targeted group for oral health education programs. School teachers have always played a diversified role in bringing multiple developmental changes in school children and premises. The current initiative to advocate changes about the role of sugars in oral health and general health through school children was carried out to curb the risk of developing non-communicable diseases right from a young age. Understanding the role of sugars by school teachers and school children can further aid in primordial and primary prevention against risk factors for NCDs, the creation of sugar smart schools, and awareness to shift from the utilization of traditional artificial sources to natural sources of sugars. Identifying the different sources of sugars and the hidden forms of sugars at an early stage can further aid in preventing diseases. The development and marketing of sugar products using health or tooth-friendly logos can also facilitate early identification of products that are good for overall and oral health.

Strength and limitations

Utilizing a validated questionnaire, a vetted training module, and the inclusion of school teachers from diversified backgrounds strengthens the findings of this study. The lack of a comparison group limits the findings of this study. Although considerable efforts were undertaken to invigilate the pre and post-evaluation, the utilization of an online medium (pre and post-questionnaire via Google forms) might have led to a biased assessment. The possibility of a knowledge questionnaire acting as an intervention cannot be denied. The reportedly higher knowledge scores might have also been because the present study was carried out in a single region, which limits the generalizability. Hence, a nationwide study on the current topic is thus further warranted; including only the knowledge domain to assess oral health limits the study findings. Despite the limitations specified, the present study assessed the efficacy of training programs on a substantial sample of school teachers via knowledge change scores on the role of sugars in oral and general health. The study further opens avenues for health and oral health programs nationally and globally.

## Conclusions

The efficacy of training programs on oral health has been widely studied; however, this study focuses on a training program on the role of sugars. The study findings conclude that a training program on the role of sugars in oral and general health effectively improved school teachers' knowledge of the role of sugars in oral and general health. This study emphasizes the need for further national-level programs to provide information regarding the role of sugars and to prevent the spread of non-communicable diseases. 

## Figures and Tables

**Figure 1:**
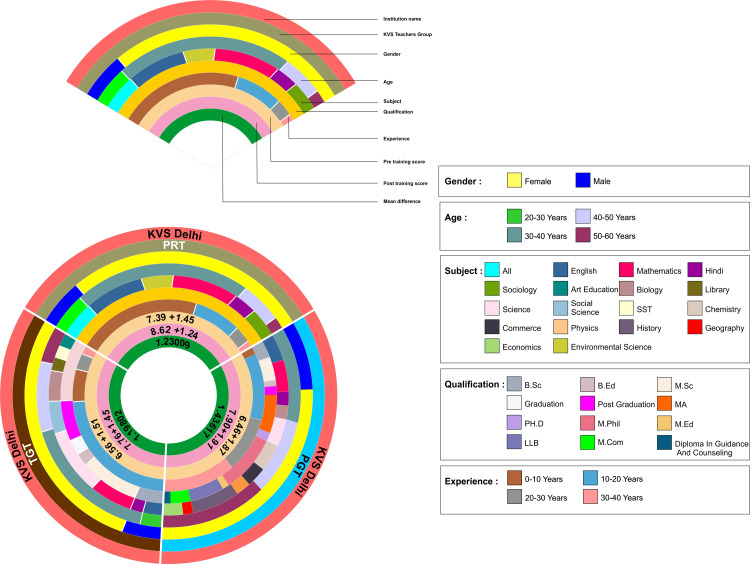
Study findings

## Appendices

The brief characteristics of the study participants and findings of this study have been shown here.
